# The Photomorphogenic Transcription Factor PpHY5 Regulates Anthocyanin Accumulation in Response to UVA and UVB Irradiation

**DOI:** 10.3389/fpls.2020.603178

**Published:** 2021-01-18

**Authors:** Yun Zhao, Ting Min, Miaojin Chen, Hongxun Wang, Changqing Zhu, Rong Jin, Andrew C. Allan, Kui Lin-Wang, Changjie Xu

**Affiliations:** ^1^Zhejiang Provincial Key Laboratory of Horticultural Plant Integrative Biology, Zhejiang University, Hangzhou, China; ^2^College of Food Science & Engineering, Wuhan Polytechnic University, Wuhan, China; ^3^Fenghua Institute of Honey Peach, Fenghua, China; ^4^College of Biology and Pharmaceutical Engineering, Wuhan Polytechnic University, Wuhan, China; ^5^New Zealand Institute for Plant & Food Research Limited, Auckland, New Zealand; ^6^School of Biological Sciences, University of Auckland, Auckland, New Zealand

**Keywords:** peach, anthocyanin, UVA, UVB, *HY5*, photoreceptor, *PpMYB10.1*

## Abstract

Red coloration contributes to fruit quality and is determined by anthocyanin content in peach (*Prunus persica*). Our previous study illustrated that anthocyanin accumulation is strongly regulated by light, and the effect of induction differs according to light quality. Here we showed that both ultraviolet-A (UVA) and ultraviolet-B (UVB) irradiation promoted anthocyanin biosynthesis in “Hujingmilu” peach fruit, and a combination of UVA and UVB had additional effects. The expression of anthocyanin biosynthesis and light signaling related genes, including transcription factor genes and light signaling elements, were induced following UV irradiation as early as 6 h post-treatment, earlier than apparent change in coloration which occurred at 72 h. To investigate the molecular mechanisms for UVA- and UVB-induced anthocyanin accumulation, the genes encoding ELONGATED HYPOCOTYL 5 (HY5), CONSTITUTIVE PHOTOMORPHOGENIC1 (COP1), Cryptochrome (CRY), and UV RESISTANCE LOCUS 8 (UVR8) in peach were isolated and characterized through functional complementation in corresponding Arabidopsis (*Arabidopsis thaliana*) mutants. *PpHY5* and *PpCOP1.1* restored hypocotyl length and anthocyanin content in Arabidopsis mutants under white light; while *PpCRY1* and *PpUVR8.1* restored *AtHY5* expression in Arabidopsis mutants in response to UV irradiation. Arabidopsis *PpHY5/hy5* transgenic lines accumulated higher amounts of anthocyanin under UV supplementation (compared with weak white light only), especially when UVA and UVB were applied together. These data indicated that PpHY5, acting as AtHY5 counterpart, was a vital regulator in UVA and UVB signaling pathway. In peach, the expression of *PpHY5* was up-regulated by UVA and UVB, and PpHY5 positively regulated both its own transcription by interacting with an E-box in its own promoter, and the transcription of the downstream anthocyanin biosynthetic genes *chalcone synthase 1* (*PpCHS1*), *chalcone synthase 2* (*PpCHS2*), and *dihydroflavonol 4-reductase* (*PpDFR1*) as well as the transcription factor gene *PpMYB10.1*. In summary, functional evidence supports the role of PpHY5 in UVA and UVB light transduction pathway controlling anthocyanin biosynthesis. In peach this is via up-regulation of expression of genes encoding biosynthetic enzymes, as well as the transcription factor *PpMYB10.1* and *PpHY5* itself.

## Introduction

Anthocyanins, a group of secondary metabolites known as flavonoid compounds, are important water-soluble pigments widely distributed in plants where they perform a multitude of biological functions such as protecting against a variety of abiotic stresses [ultraviolet (UV) radiation, nutrient deficiency, low temperature, drought, etc.], attracting pollinators and seed dispersers, and defense against pathogens and herbivores (Schaefer et al., 2004; Gould et al., 2010). In addition, they are also recognized to have potential human health benefits in aspects such as prevention from cancer and heart disease (Tsuda, 2012; Zhang et al., 2015). Hence, anthocyanin is considered as an essential determinant for fruit quality.

The anthocyanin biosynthetic pathway has been extensively studied and found to be relatively conserved among plant species (Winkel-Shirley, 2001). Anthocyanins are biosynthesized via the phenylpropanoid pathway, which is catalyzed stepwise by a series of enzymes including phenylalanine ammonia lyase (PAL), cinnamic acid-4-hydroxylase (C4H), 4-coumarate CoA ligase (4CL), chalcone synthase (CHS), chalcone isomerase (CHI), flavanone 3-hydroxylase (F3H), flavonoid 3’-hydroxylase (F3’H), flavonoid 3’,5’-hydroxylase (F3’5’H), dihydroflavonol 4-reductase (DFR), anthocyanidin synthase (ANS), and UDP-glucose: flavonoid 3-*O*-glucosyltransferase (UFGT; Jaakola, 2013; Zhang et al., 2014). The expression of the genes that encode these enzymes are transcriptionally regulated by MYB, basic Helix-Loop-Helix (bHLH), and WD40 repeat proteins, often in a form of trimeric protein complex of MYB-bHLH-WD40 (the MBW complex; Lin-Wang et al., 2010; BenSimhon et al., 2011; An et al., 2012; Chagné et al., 2013; Wang N. et al., 2018). In peach (*Prunus persica*), several relevant transcription factors (PpMYB10s, PpbHLH3, and PpWD40-1) have been functionally confirmed to control anthocyanin biosynthesis (Ravaglia et al., 2013; Rahim et al., 2014; Uematsu et al., 2014; Zhou et al., 2014, 2018; Tuan et al., 2015).

Anthocyanin biosynthesis is not only developmentally regulated but also influenced by various environmental and endogenous stimuli as well (Guo et al., 2008). As the primary energy source and an informational signal for regulating plant growth and development, light is one of the most critical environmental factors (Kami et al., 2010). Light profoundly influences complex signaling pathways such as photomorphogenesis, circadian rhythms and the regulation of secondary metabolism throughout the life of plants (Gelderen et al., 2018).

Previous research has revealed the significance of light on the induction of anthocyanin biosynthesis in fruits such as bilberry (*Vaccinium myrtillus*, Uleberg et al., 2012), Chinese bayberry (*Myrica rubra*, Niu et al., 2010), grapes (*Vitis vinifera*, Azuma et al., 2012), raspberry (*Rubus idaeus*, Wang et al., 2009) and cranberry (*Vaccinium macrocarpon*, Zhou and Singh, 2004), etc. The effects of light on anthocyanin accumulation vary with different light wavelengths, where UV light always presents a more profound effect on anthocyanin biosynthesis compared with white light (Zoratti et al., 2014). For instance, ultraviolet-B (UVB) irradiation significantly increased anthocyanin content in the peel of apple (*Malus domestica*) and pear (*Pyrus pyrifolia*) fruits (Hagen et al., 2007; Qian et al., 2013; Sun et al., 2014; Henry-Kirk et al., 2018). Tomato (*Solanum lycopersicum*) grown under ultraviolet-A (UVA) supplementation visibly accumulated more anthocyanin in a short time (Guo and Wang, 2010; Castagna et al., 2014). In strawberries (*Fragaria ananassa*), blue light and UVC irradiation stimulated anthocyanin accumulation and enhanced the antioxidant activities (Li et al., 2014; Xu et al., 2014). In peach, it has also been demonstrated that UV lights are stronger stimulants for anthocyanin accumulation (Zhao et al., 2017).

Higher plants have evolved extremely advanced systems to sense changes in ambient light conditions (Casal, 2013), by being equipped with specialized sensory photoreceptors for specific wavelengths of light, including red/far-red light receptor phytochromes (PHYA to PHYE) (Oh and Montgomery, 2017), blue/UVA light receptor cryptochromes (CRY1 and CRY2) or phototropins (PHOTs; Lin, 2002; Liu et al., 2011; Wang Q. et al., 2018), and UVB light receptor UV RESISTANCE LOCUS 8 (UVR8; Rizzini et al., 2011; Yin and Ulm, 2017; Liang et al., 2019). Photoreceptors are indispensable for light-induced anthocyanin biosynthesis (Yang et al., 2016), as in Arabidopsis (*Arabidopsis thaliana*), the photoreceptor deficient mutants *uvr8* and *cry1cry2* respectively have a weak response in UVB and blue light/UVA-induced photomorphogenesis like blocking the inhibition of hypocotyl elongation and the induction of anthocyanin biosynthesis (Wade et al., 2001; Morales et al., 2013; Wang Q. et al., 2018).

After perceiving light, this signal is transmitted from photoreceptors to downstream light signal transduction factors to regulate the process of light-induced anthocyanin biosynthesis both transcriptionally and post-translationally (Casal, 2013). ELONGATED HYPOCOTYL 5 (HY5), a basic leucine zipper (bZIP) transcription factor, is a master regulator in light signal transduction pathway that acts downstream of multiple photoreceptors to respond to photomorphogenesis (Holm et al., 2002; Gangappa and Botto, 2016). HY5 is considered as a positive regulator to participate in light-induced anthocyanin biosynthesis in Arabidopsis, apple, grape, and pear (Loyola et al., 2016; An et al., 2017; Nawkar et al., 2017; Tao et al., 2018). AtHY5 targets the anthocyanin biosynthetic genes (*CHS*, *F3H*) and *MYB* [*PRODUCTION OF FLAVONOL GLYCO-SIDES 1* (*PFG1*)/*MYB12*, *MYB75*/*PRODUCTION OF ANTHOCYANIN PIGMENT* 1 (*PAP1*)] to activate their expression by direct binding to the promoters (Chattopadhyay et al., 1998; Lee et al., 2007; Stracke et al., 2010; Shin et al., 2013). In eggplant (*Solanum melongena*) under blue light, SmHY5 has also been revealed to accelerate anthocyanin accumulation by regulating the expression of downstream genes encoding enzymes of the anthocyanin biosynthetic pathway (Jiang et al., 2016). In apple, MdHY5 promoted the expression of *MdMYB10* to regulate anthocyanin accumulation (An et al., 2017). CONSTITUTIVE PHOTOMORPHOGENIC1 (COP1), an E3 ubiquitin ligase, is a crucial repressor for photomorphogenesis and anthocyanin biosynthesis, targeting HY5 for proteasome-mediated degradation (Osterlund et al., 2000; Lau and Deng, 2012). In pear, PpyCOP1 indirectly interacted with PpyHY5 to mediate regulation of anthocyanin accumulation (Tao et al., 2018). Additionally, apple MdCOP1 was also found to be critical for the ubiquitination and degradation of MdMYB1, a positive regulator for anthocyanin biosynthesis, further leading to the negative regulation for the coloration in apple fruit (Li et al., 2012).

Anthocyanin accumulation is essential for the quality of peach fruit. Our previous study revealed that UV light enhances anthocyanin content in peach peel (Zhao et al., 2017). However, how photoreceptors and light signal transduction elements mediate UV light-induced anthocyanin accumulation has not been elucidated. In this study, the effect of UVA and UVB irradiation on the coloration of peach fruit was examined. We cloned and functionally characterized *PpHY5* and provided molecular evidence that PpHY5 plays a vital role in anthocyanin biosynthesis in response to UVA and UVB. These results contributed to our understanding of the mechanism underlying UV light-induced anthocyanin biosynthesis in peach, as well as providing evidence for improving fruit pigmentation.

## Materials and Methods

### Plant Materials and Growth Conditions

The peach cultivar (cv) “Hujingmilu” was used in this study. All fruit were bagged with commercial yellow paper bags at 42 days after full blossom and collected at just before turning stage. For UV irradiation treatment experiments, all collected fruit were transferred to a growth chamber at a constant 20°C and irradiated with continuous UVA (320–400 nm, 1,000 μw/cm^2^) or UVB (280–320 nm, 58 μw/cm^2^) or both together for 0, 6, 12, 24, 48, 72, and 144 h, respectively. The fruit kept in the dark served as the control. Peel tissue from 30 fruit of each treatment were separated simultaneously into three biological replicates (10 fruit for each replicate) at every sampling time, then immediately frozen in liquid nitrogen and stored at −80°C for subsequent experiments.

The Arabidopsis *hy5* (SALK_056405), *cop1* (SALK_022133), *uvr8* (SALK_033468), and *cry1* (SALK_069292) mutants with the Columbia genetic background were obtained from The Arabidopsis Information Resource (TAIR). For hypocotyl length and anthocyanin accumulation assays, seeds of wild type (WT), above-mentioned mutants as well as transgenic lines obtained in this study were sterilized and germinated on Murashige and Skoog plates supplemented with 0.6% agar and 1% sucrose, and subjected to a chilling treatment at 4°C for 2 days in the dark. Then these seedlings were transferred to a growth chamber at 24°C under the long-day condition (16 h photoperiod/8 h dark cycle) of white light in normal intensity (40 w/m^2^) for growth. For UV light treatment experiment, seedlings were grown under continuous weak white light (WWL, 40 μw/cm^2^) supplemented with UVA (40 μw/cm^2^) or UVB (10 μw/cm^2^) or both together at 24°C. And the seedlings kept under only WWL served as the control. After growth under different light conditions as indicated for 5 or 7 days, respectively, the hypocotyl length was measured and seedlings were sampled for subsequent experiments.

### Color Measurement

Fruit surface color measurement was carried out using a reflectance spectrophotometer (Hunter Lab Mini Scan XE Plus colorimeter) at four evenly distributed equatorial positions of per fruit. The raw data as L^∗^ (lightness, from black to white), a^∗^ (the degree of green-red variation), b^∗^ (the degree of blue-yellow variation) values were recorded and the color index of red grapes (CIRG) was calculated according to the formula CIRG = (180 − H)/(L^∗^ + C), while H = arctan (b^∗^/a^∗^) and C = [(a^∗^)^2^+(b^∗^)^2^]^0.5^ (Carreño et al., 1995; Zhang et al., 2008).

### Anthocyanin Extraction and Measurement

Anthocyanin was extracted from peach fruit peel and Arabidopsis seedlings by using the method as described in our previous study (Zhao et al., 2017). High-performance liquid chromatography (HPLC) analysis for quantification of anthocyanin was performed using an Agilent 1260 liquid chromatograph system (Agilent Technologies, CA, United States) equipped with a ZORBAX SB-C18 column following a previously reported protocol (Cheng et al., 2014). The UV-visible light detector wavelength was set at 520 nm. Cyanidin 3-*O*-glucoside chloride was employed as the authentic standard.

### RNA Extraction and Reverse Transcription Quantitative PCR Analysis

Total RNA was isolated from peach tissue samples and Arabidopsis by using a modified cetyltrimethylammonium bromide (CTAB) method (Liu et al., 2015) and the TRIzol Reagent Kit (Ambion, Hopkinton, MA, United States), respectively. First-strand cDNA was synthesized with HiScript^®^ II Q Select RT SuperMix (Vazyme) after removal of genomic DNA contamination by TURBO DNase (Ambion) following the manufacturer’s protocol. Reverse transcription quantitative PCR (RT-qPCR) was performed using the SsoFast EvaGreen Supermix kit (Bio-Rad, CA, United States) with the CFX96 instrument (Bio-Rad, CA, United States) according to the following program: 95°C for 3 min, followed by 45 cycles of 95°C for 10 s, 60°C for 30 s and then 95°C for 10 s followed by a continuous increase from 65°C to 95°C at the heating rate of 0.5°C/s for dissociation curve analysis (Zhao et al., 2017). The *PpTEF2* (GenBank accession: No. JQ732180) and *ATACT2* (GenBank accession: No. AT3G18780) genes were used as the internal reference genes to normalize expression values for peach and Arabidopsis, respectively (Tong et al., 2009; Zhou et al., 2013). The primer sequences for RT-qPCR were designed with Primer Premier 5 and listed in Supplementary Table 1.

### RNA-Seq Analysis

RNA sequencing (RNA-Seq) of peach fruit samples (irradiated with different UV-light conditions for 0, 6, 12, 24, and 48 h) was conducted using the Xten platform by staff at Biomarker (Beijing, China) with three biological replicates for each sample. Data analysis was conducted as described in our previous work (Zhao et al., 2017). Transcript abundances were normalized by calculating FPKM (expected number of Fragments Per Kilobase of transcript sequence per Million base pairs sequenced).

### Plasmid Construction

The full-length coding sequence of *PpHY5* (Prupe.1G478400), *PpHYH* (Prupe.1G208500), *PpCOP1.1* (Prupe.5G031300), *PpUVR8*.*1* (Prupe.4G277200), *PpCRY1* (Prupe.1G517600) was isolated and recombined into the pGreenII 0029 62-SK vector (*PpHY5*-SK, *PpHYH*-SK, *PpCOP1*.*1*-SK, *PpUVR8.1*-SK, *PpCRY1*-SK). The promoter sequence (1500 bp upstream of the initiation codon) of structural genes (*PpCHS1*, *PpCHS2*, *PpCHI*, *PpF3H*, *PpF3’H*, *PpDFR*, *PpANS*, and *PpUFGT*) and transcription factors (*PpMYB10.1*/*2*/*3*) were constructed into the pGreen II 0800-LUC vector (*PpCHS1*-LUC, *PpCHS2*-LUC, *PpCHI*-LUC, *PpF3H*-LUC, *PpF3’H*-LUC, *PpDFR*-LUC, *PpANS*-LUC, *PpUFGT*-LUC, and *PpMYB10*.*1*/*2*/*3*-LUC). In experiments for verifying the function of E-box motifs in *PpHY5* promoter, mutations on the *PpHY5* promoter were conducted using the Fast Mutagenesis System Kit (Transgene) and inserted into the pGreenII 0800-LUC vector, named *PpHY5m1*-LUC, *PpHY5m2*-LUC, *PpHY5m3*-LUC, and *PpHY5m4*-LUC, respectively. For subcellular localization analysis, the coding sequence of each gene without stop codon was fused with green fluorescent protein (GFP) and cloned into the pCAMBIA super 1300-eGFP vector (*PpHY5*-GFP, *PpCOP1.1*-GFP). All constructs were transformed into *Agrobacterium tumefaciens* strain GV3101 (MP90) by electroporation with GenePulser Xcell^TM^ Electroporation Systems (Bio-Rad, Hercules, CA, United States) for subsequent experiments. Primers used for plasmid construction were described as listed in Supplementary Table 2.

### Dual-Luciferase Assay

The dual-luciferase assay was performed with *Nicotiana benthamiana* leaves by using a previously reported protocol (Zhao et al., 2020). *Agrobacterium* cultures containing recombinant pGreenII 0029 62-SK vectors harboring transcription factor genes and the cultures containing recombinant pGreen II 0800-LUC vector harboring target gene promoters were infiltrated into *N*. *benthamiana* leaves with a ratio of 10:1 (v/v). At three days following infiltration, the firefly luciferase (LUC) and renilla luciferase (REN) activities were measured using dual-Luciferase Reporter Assay System (Promega, MI, United States) with Modulus Luminometer. When the ratio of LUC/REN was over two and the difference was highly significant (*P* < 0.01) as compared with the control, the transcription factor was considered to be able to activate the promoter. For each transcription factor and promoter interaction, dual-luciferase assays were conducted with at least three independent experiments (six replicate reactions in each experiment).

### Arabidopsis Transformation and Hypocotyl Length Measurement

The Arabidopsis transformation was performed by using the floral dip method (Zhang et al., 2006). SilwetL-77 (Real-Times, Beijing, China) was applied as a surfactant in *Agrobacterium*-based transformation of Arabidopsis. Transgenic seeds were sterilized and placed on Murashige and Skoog medium containing 50 mg/mL kanamycin for transgenic plant selection. For each target gene, at least three lines were characterized and three of them with highest expression levels of target gene were selected, and individuals of the T2 were used for further phenotype analysis. After growth for 5 or 7 days, the hypocotyl length measurement of at least 30 Arabidopsis seedlings was conducted for each replicate. All experiments were carried out with three independent biological replicates.

### Subcellular Localization Analysis

The *Agrobacterium* strain carrying the 35S::*PpHY5*-GFP or 35S::*PpCOP1.1*-GFP recombinant vector was infiltrated into transgenic *N*. *benthamiana* plant with a red fluorescent nuclear marker (Nucleus-RFP) according to a previous report (Zhao et al., 2020), while 35S::GFP was used as a negative control. The GFP florescence of the transiently expressed leave was observed under a Zeiss LSM710NLO confocal laser scanning microscope.

### *Cis*-regulatory Element Analysis

The predictions of conserved *cis*-regulatory elements in the promoter were analyzed using the PlantCARE database.^1^

### Statistical Analysis

The statistical significance of differences was analyzed using SPSS statistical software (SPSS 19.0, SPSS Inc., Chicago, IL, United States) with Student’s *t*-test. Graphs were plotted using Origin 8.0 (Origin Lab Corporation, Northampton, MA, United States).

## Results

### Time Course of Color Development and the Expression of Anthocyanin Biosynthetic Genes Under Continuous UV Treatments

**FIGURE 1:**
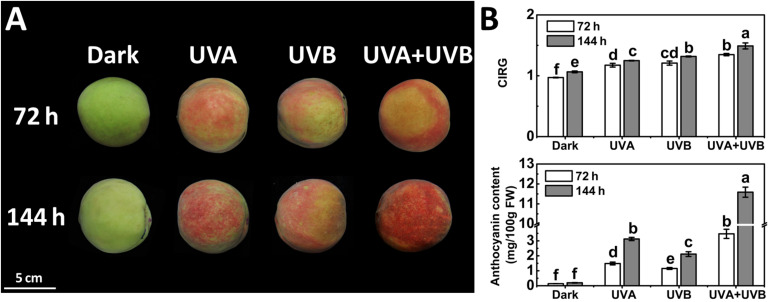
Coloration **(A)** and the color index of red grapes (CIRG) values and anthocyanin content **(B)** in the peach peel after continuous UV irradiation or kept in dark. UVA, 1,000 μw/cm^2^; UVB, 58 μw/cm^2^. Scale bar indicated 5 cm. Data were presented as means (±SE) from three independent biological replicates. Values labeled with different letters indicated a significant difference at *P* < 0.05.

To investigate the influence of different UV light treatments on anthocyanin biosynthesis in peach, the fruit were given either a continuous UVA or UVB or UVA + UVB treatment. The control fruit was kept in the dark and did not develop red color over time. Under UV irradiation, the peel color remained green within 24 h and a few red spots began to be slightly visible at 48 h. At 72 and 144 h following irradiation, the fruit turned deep red (Figure 1A). These observations matched well with the CIRG values (Figure 1B). Under dark condition, the concentration of anthocyanin in peel was very low and showed no significant increase over time (Figure 1B). In contrast, anthocyanin accumulated following irradiation with UVA or UVB or UVA + UVB, and the effects varied significantly among different UV lights conditions. The concentration of anthocyanin rose sharply to 3.12 mg/100gFW (15-fold higher than the control) under UVA exposure and 2.11 mg/100gFW (10-fold higher than the control) under UVB exposure at 144 h respectively. During the irradiation period, the highest anthocyanin content was detected in fruit under UVA + UVB treatment, reaching 3.47 mg/100gFW at 72 h (2–3 fold higher than UVA or UVB) and 11.59 mg/100gFW at 144 h (3-6 fold higher than UVA or UVB), respectively (Figure 1B), which was consistent with the visual appearance.

**FIGURE 2:**
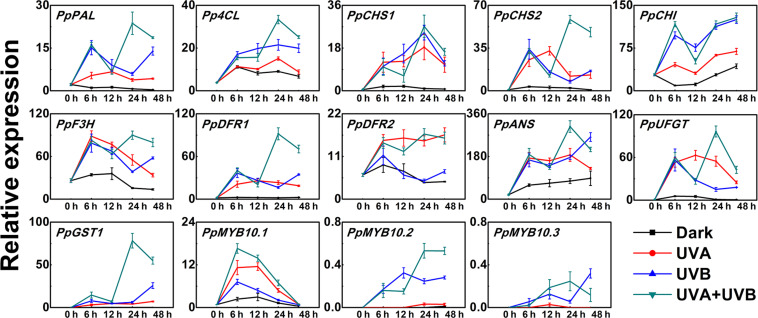
Time course of the transcript levels of anthocyanin biosynthesis related genes in peach peel in response to continuous UV lights. UVA, 1,000 μw/cm^2^; UVB, 58 μw/cm^2^. Data were presented as means (±SE) from three independent biological replicates.

In order to further explore the molecular mechanisms for anthocyanin accumulation in response to UV lights, we analyzed the transcript levels of genes encoding anthocyanin biosynthetic enzymes (*PpPAL*, *Pp4CL*, *PpCHS1*, *PpCHS2*, *PpCHI*, *PpF3H*, *PpDFR1*, *PpDFR2*, *PpANS*, and *PpUFGT*), transport genes *PpGST1* and regulatory genes (*PpMYB10.1*, *PpMYB10.2*, and *PpMYB10.3*) in peel during the early irradiation period (0, 6, 12, 24, and 48 h) before the appearance of red coloration. Under dark condition, the assayed genes exhibited low expression levels and for some genes such as *PpCHS1*, *PpDFR1*, and *PpUFGT*, the transcript levels showed little change over time (Figure 2). In comparison to the low expression levels at the initial point (0 h), the expression profiles of the biosynthetic genes were up-regulated to various degrees in peel of fruit when exposed to the UV treatments. The transcripts of all assayed biosynthetic genes under different UV light conditions were elevated than control (Figure 2). After exposure to UV light for 6 h, the expression levels of all analyzed biosynthetic genes sharply increased (Figure 2). For example, the transcript levels of *PpCHS1* and *PpUFGT* in the UV light-exposed fruit were at least 6-fold and 9-fold higher than the control. However, after exposure to UV light for 12 h, the transcript levels of *PpCHI*, *PpF3H*, *PpANS*, and *PpGST1* decreased slightly. *Pp4CL* and *PpCHS1* showed the highest expression after 24 h of UV light irradiation. Although there was a slight decline in the transcript abundance of some genes after irradiation for 48 h, the expression was consistently higher than the control, for instance, the transcript levels of *PpDFR1* were at least 7-fold higher, 14-fold higher and 28-fold higher in the UV light-exposed fruit than that in the dark, respectively. In particular, the expression of *PpGST1* continuously remained very low in the fruit under dark condition, while was strongly stimulated by UV light, showing a 43-fold, 155-fold, and 332-fold elevation at 48 h in UVA, UVB, and UVA + UVB light exposed fruit, respectively.

The expression patterns of the regulatory transcription factor genes (*PpMYB10.1*, *PpMYB10.2*, and *PpMYB10.3*) showed elevation by UV, but with different profiles. The transcript level of *PpMYB10.1* was strongly induced by UV light and then decreased after prolonged exposure. The expression of *PpMYB10.2* and *PpMYB10.3* presented little change under UVA irradiation and transcript abundance was lower compared with *PpMYB10.1* (Figure 2). The transcript levels of most genes were generally highest in peel of fruit irradiated with UVA + UVB, followed by UVA and UVB, while the expression were almost undetectable or showed low levels in control. Even though there were slight declines in transcription level at some time points, most genes reached the maximum transcription levels when treated with UVA + UVB for 24 h, which ensured the high transcription abundance detected in fruit at later stages. The results suggested that the coloration of the peel was able to be induced by the irradiation of UVA or UVB, and there was a synergistic effect of UVA and UVB in stimulating anthocyanin biosynthesis.

### Expression Analysis of *PpHY5* and *PpCOP1.1* after UV Light Treatment

In Arabidopsis, it has been demonstrated that *HY5* and *COP1* play vital roles in light signal transduction pathway (Lau and Deng, 2012; Gangappa and Botto, 2016). Previously, we identified potential homologous genes in peach (named *PpHY5*, *PpHYH*, *PpCOP1*.*1*, and *PpCOP1.2*) (Zhao et al., 2017). To clarify their functions in UV light induced anthocyanin biosynthesis in peach fruit, the expression levels following UV light exposure were monitored.

**FIGURE 3:**
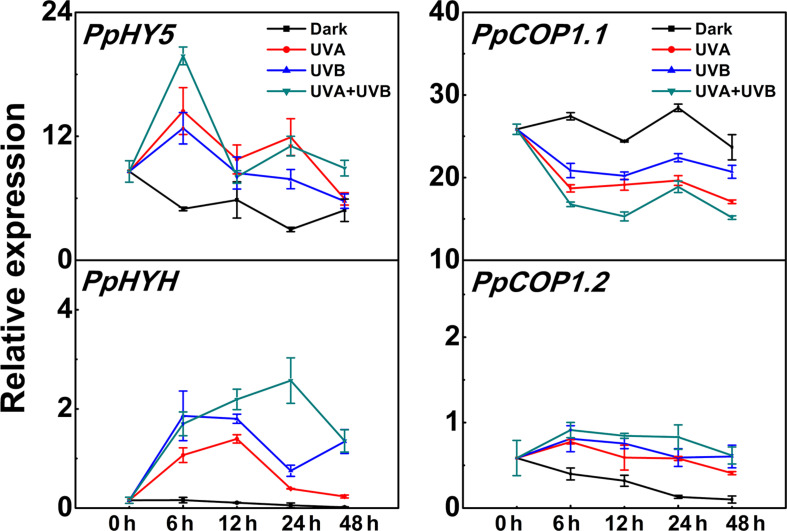
Time course of the transcript levels of *PpHY5*, *PpHYH* and *PpCOP1.1*, *PpCOP1.2* in peach peel in response to continuous UV lights. UVA, 1,000 μw/cm^2^; UVB, 58 μw/cm^2^. Data were presented as means (±SE) from three independent biological replicates.

*PpHY5* expression exhibited a strong response to UV light irradiation (Figure 3). The transcript level of *PpHY5* was low at the beginning (0 h); with UV there was a sharp increase which peaked within the first 6 h, and showed the highest accumulation level under UVA + UVB (about 4-fold higher than the control); from this peak there was a gradual decrease but expression remained higher than control (Figure 3). In Arabidopsis, HY5 HOMOLOG (HYH) has multiple overlapping functions with HY5 (Holm et al., 2002). In our study, the transcript level of *PpHYH* was similar to those of *PpHY5*, which increased at the beginning of irradiation but eventually decreased with continuous UV light (Figure 3). By contrast, the expression of *PpCOP1*.*1* decreased gradually following irradiation with UVA or UVB or UVA + UVB while slightly increased in the control (Figure 3). However, *PpCOP1.2* remained at quite low levels of expression with no obvious variation during the entire irradiation period among all different treatments (Figure 3). Overall, expression of *PpHY5* and *PpHYH* increased following UV treatment while that of *PpCOP1.1* was reduced. The transcript levels of these genes also responded quickly, within 6 h after the start of the treatment.

### *PpHY5* and *PpCOP1.1* Restored Hypocotyl Length and Anthocyanin Content in Arabidopsis Mutants under White Light

To confirm the roles of *PpHY5* and *PpCOP1.1* as light signal transduction related genes, we conducted the functional complementation assays with the Arabidopsis *hy5* mutant and *cop1* mutant transformed with *PpHY5* and *PpCOP1.1*, respectively. At least three independent transgenic lines of each background were generated and confirmed by RT-qPCR. The transgenic lines (*PpHY5*/*hy5*, *PpCOP1.1*/*cop1*) with high expression of *PpHY5* or *PpCOP1.1* were chosen to compare with WT and *hy5* or *cop1* mutant by measuring hypocotyl length and anthocyanin content under all conditions in 5-day-old seedlings.

**FIGURE 4:**
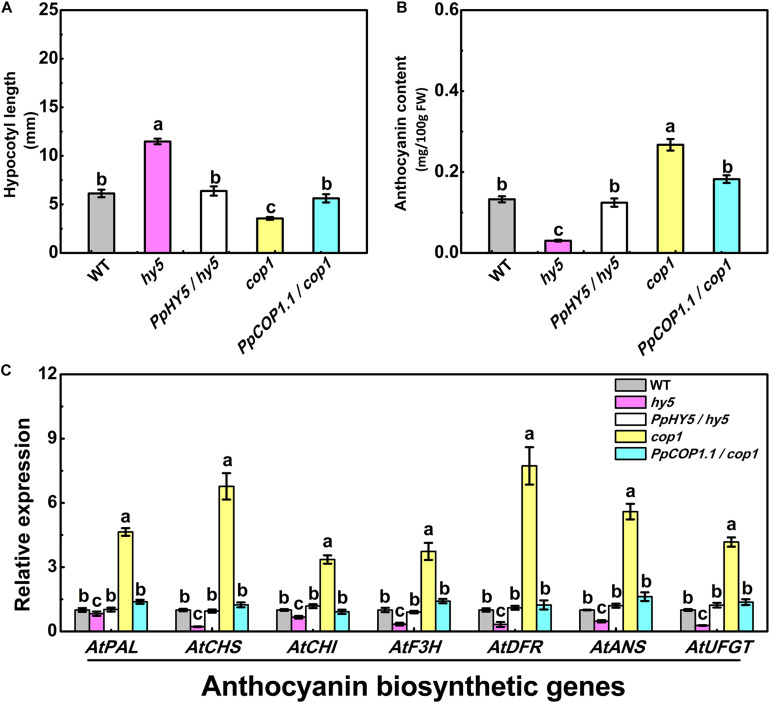
Hypocotyl length **(A)**, anthocyanin content **(B)** and the relative expression levels of anthocyanin biosynthesis related genes **(C)** in 5-day-old seedlings of wild type (WT), mutant (*hy5*, *cop1*) and transgenic plants (*PpHY5*/*hy5*, *PpCOP1.1*/*cop1*) grown under long-day condition (16 h 40 w/m^2^ white light/8 h dark cycle). For Arabidopsis transgenic plants, three independent lines for each target gene were used. Data were presented as means (±SE) from three independent biological replicates with 30 or more Arabidopsis seedlings collected for each replicate. Values labeled with different letters indicated a significant difference at *P* < 0.05.

For plants grown under white light for five days, *hy5* exhibited the longest hypocotyl phenotype (11.47 mm), while *cop1* exhibited the shortest hypocotyl phenotype (3.55 mm) (Figure 4A). The constitutive overexpression of *PpHY5* or *PpCOP1.1* in corresponding mutant background restored the hypocotyl length under white light (Figure 4A). The concentrations of anthocyanin in all lines also showed difference to some degrees (Figure 4B). Compared to WT, *cop1* displayed significantly higher accumulation of anthocyanin, and a lower level was observed in *hy5*. The anthocyanin levels were restored and enhanced in *PpHY5*/*hy5* transgenic lines, reaching a level that was about 4.1-fold higher than that in *hy5* mutant background (Figure 4B). In comparison, the anthocyanin accumulation was inhibited in the *PpCOP1.1*-complemented lines and decreased by 32% than those in *cop1* mutant background (Figure 4B). Likewise, the genes encoding enzymes of the anthocyanin biosynthetic pathway (*PAL*, *CHS*, *CHI*, *F3H*, *DFR*, *ANS*, and *UFGT*) exhibited similar transcript accumulation trends consistent with anthocyanin content (Figure 4C). All these structural genes were generally lowly expressed in *hy5* and highly expressed in *cop1* relative to WT. For instance, the expression levels of *ANS* and *UFGT* decreased significantly in *hy5* and were about 50 and 75% lower, whereas at least 5-fold and 4-fold higher in *cop1* than that measured in WT. No significant differences in transcript levels of anthocyanin biosynthetic structural genes were found between *PpHY5*/*hy5* or *PpCOP1*.*1*/*cop1* transgenic lines and WT.

To visualize the subcellular localization of PpHY5 and PpCOP1.1, we performed the confocal microscopy assays of *N*. *benthamiana* leaves transiently transformed with *PpHY5*-GFP and *PpCOP1.1*-GFP. Our results showed that both PpHY5-GFP and PpCOP1.1-GFP displayed signal in the nucleus under white light (Supplementary Figure 1). This was in consistent with the subcellular localization of AtHY5 and AtCOP1 (Hardtke et al., 2000; Osterlund et al., 2000; Lau and Deng, 2012). These data suggested that *PpHY5* and *PpCOP1.1* were the functional orthologous of *AtHY5* and *AtCOP1* and were able to participate in white-light-related photomorphogenesis.

### *PpHY5* Participated in UVA- and UVB-Induced Anthocyanin Accumulation in a *PpCRY1* and *PpUVR8.1* Dependent Manner

**FIGURE 5:**
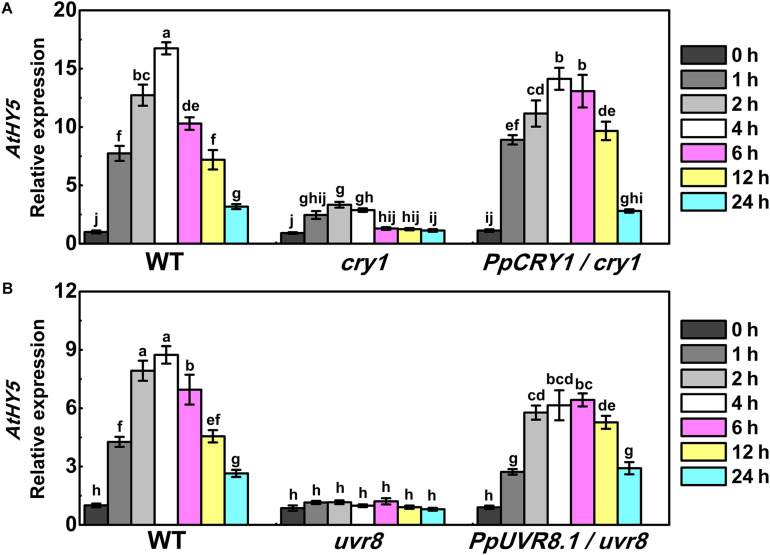
The expression of *AtHY5* in the wild type (WT), *cry1* mutant and *PpCRY1*/*cry1* transgenic plant with different durations of continuous weak white light (WWL, 40 μw/cm^2^) + UVA irradiation (40 μw/cm^2^) **(A)** or in the WT, *uvr8* mutant and *PpUVR8*.*1*/*uvr8* transgenic plant with different durations of continuous WWL (40 μw/cm^2^) + UVB irradiation (10 μw/cm^2^) **(B)**. For Arabidopsis transgenic plants, three independent lines for each target gene were used. 5-day-old seedlings grown under constant WWL were subjected to WWL + UVA or WWL + UVB irradiation for the indicated time span. Data were presented as means (±SE) from three independent biological replicates with 30 or more Arabidopsis seedlings collected for each replicate.

In Arabidopsis, AtHY5 is a key factor in photomorphogenic responses and acts as a downstream signaling intermediate of AtCRY1 and AtUVR8 (Binkert et al., 2014; Marzi et al., 2020). To determine whether the peach counterpart of these genes function in the same signaling cascade in Arabidopsis, we generated the *PpHY5*/*hy5*, *PpCRY1/cry1*, and *PpUVR8.1*/*uvr8* transgenic lines. Five-day-old seedlings of WT, *cry1* mutant and *PpCRY1*/*cry1* transgenic lines were exposed to WWL + UVA for periods of 0–24 h and the expression of *AtHY5* was examined. Upon WWL + UVA exposure, the transcript levels of *AtHY5* were up-regulated and reached the maximum at 4 h (the upregulation ratio of *PpCRY1*/*cry1* was 13-fold, for *cry1* mutant it was 3-fold, for WT it was 16-fold), then a decreased occurred with the increment of irradiation time (Figure 5A). In comparison, *AtHY5* in *PpCRY1*-overexpression seedling in *cry1* background exhibited a similar transcript accumulation pattern as WT which was consistently higher than *cry1* mutant. With complementation of the *cry1* mutant, it can be concluded that *PpCRY1* was a functional counterpart of *AtCRY1* and could regulate *AtHY5* expression in response to UVA irradiation.

Similarly, after five days growth under white light, WT, *uvr8* mutant and *PpUVR8.1*/*uvr8* transgenic lines were transferred to WWL + UVB condition for periods of 0-24 h. Before WWL + UVB irradiation (0 h), *AtHY5* displayed relatively low transcription levels in all lines (Figure 5B). After exposure to WWL + UVB, a UV-induced elevation in *AtHY5* transcription occurred in WT, while no increase was observed in *uvr8*. However, the overexpression of *PpUVR8.1* in *uvr8* restored the ability of UVB to increase *AtHY5* transcription. The transcript levels of *AtHY5* showed a similar pattern in seedlings of *PpUVR8.1*/*uvr8* transgenic lines and WT during the irradiation period, decreasing gradually after a rapid peaking within 4 h. This suggested that the *PpUVR8.1* was a functional counterpart of *AtUVR8* and could regulate the expression of *AtHY5* in response to UVB irradiation.

**FIGURE 6:**
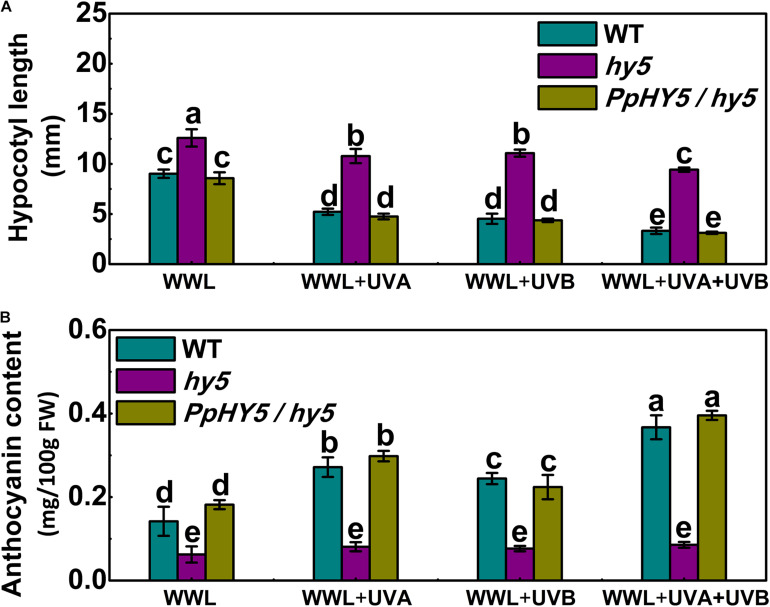
Effect of different light treatments on hypocotyl length **(A)** and anthocyanin content **(B)** of 7-day-old seedlings of wild type (WT), *hy5* mutant and *PpHY5*/*hy5* transgenic plant. Seedlings were grown under continuous weak white light (WWL, 40 μw/cm^2^) supplemented with/without UVA (40 μw/cm^2^) or UVB (10 μw/cm^2^) or both together for different UV light treatments. For Arabidopsis transgenic plant, three independent lines were used. Data were presented as means (±SE) from three independent biological replicates with 30 or more Arabidopsis seedlings collected for each replicate. Values labeled with different letters indicated a significant difference at *P* < 0.05.

As described above, *AtHY5* participates in both UVA and UVB signaling pathways in the *PpCRY1* and *PpUVR8.1* dependent manner respectively, leading to regulation of hypocotyl elongation and anthocyanin biosynthesis. The *PpHY5*/*hy5* transgenic line was used for measuring hypocotyl length and anthocyanin content under WWL, WWL + UVA, WWL + UVB or WWL + UVA + UVB condition. Among *hy5*, WT and *PpHY5*/*hy5* transgenic line under different light conditions, *hy5* consistently showed the longest hypocotyl length and the lowest anthocyanin content relative to other plants (Figure 6). When overexpressing *PpHY5* in *hy5*, the phenotypes of inhibition of hypocotyl elongation and anthocyanin accumulation were restored to levels in WT. Upon UV light exposure, the hypocotyl length was suppressed and anthocyanin increased in *PpHY5*/*hy5* transgenic line and WT as compared with those under WWL, but such phenotypes of photomorphogenesis were compromised in *hy5* (Figure 6). A more profound effect on photomorphogenesis was observed when irradiated with both UVA and UVB (Figure 6). The observation was consistent with the result in peach fruit under UV light treatments, indicating that PpHY5 played a similar role to AtHY5, which directly participated in UVA- and UVB-induced photomorphogenic responses and acted as a central positive regulator downstream of PpCRY1 and PpUVR8.1 photoreceptors.

### PpHY5 Activated the Expression of Anthocyanin Biosynthetic Genes, *PpMYB10.1*, and Its Own Expression

Previous studies have demonstrated that Arabidopsis HY5 binds to the G-box (CACGTG) and ACE (ACGT containing element) motifs in the promoters of target genes in response to light (Lee et al., 2007). In Arabidopsis *PpHY5*/*hy5* transgenic lines, it was found that PpHY5 promoted the expression of anthocyanin biosynthetic structural genes (Figure 4C). In order to elucidate the function of PpHY5 in the regulation of anthocyanin accumulation in peach, the promoter regions (1,500 bp length) of anthocyanin biosynthetic genes and *PpMYB10s* were isolated.

**FIGURE 7:**
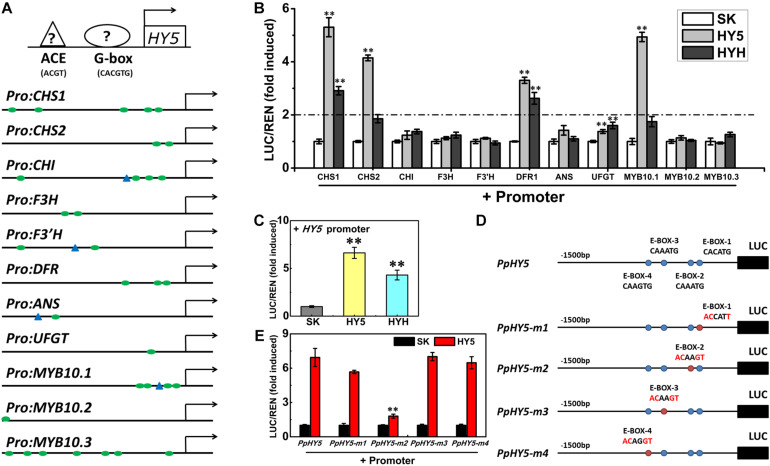
Activation/self-activation of anthocyanin biosynthesis related genes by PpHY5 or PpHYH. **(A)** Upstream sequences (1500 bp) of anthocyanin biosynthesis related genes with G-box and ACGT containing element (ACE) motifs presented as green circles and blue triangles in the promoters, respectively. **(B)** Effects of PpHY5 or PpHYH on the promoter activity of anthocyanin biosynthesis related genes in dual-luciferase assays. **(C)** Effects of PpHY5 or PpHYH on the promoter activity of *PpHY5* in dual-luciferase assays. **(D,E)** Schematic diagrams of motif mutations for the *PpHY5* promoter **(D)** and effects of PpHY5 on the activity of original and mutated promoters in dual-luciferase assays **(E)**. The ratio of LUC/REN of the empty vector (SK) plus promoter was used as the calibrator (set as 1). LUC: firefly luciferase; REN: renilla luciferase; green circles: G-box; blue triangles: ACE motif; blue circles: E-box; red circles: mutated E-box. Data were presented as means (±SE) from six independent biological replicates and Student’s *t*-test was used for statistical analyses compared with corresponding control. Asterisks (^∗∗^) indicated a highly significant difference at *P* < 0.01.

Analysis of promoter sequences revealed the presence of putative *cis*-regulatory elements related to light signaling. The HY5-binding motifs (G-box and/or ACE motifs) were distributed in the promoters of 11 genes (*PpCHS1*, *PpCHS2*, *PpCHI*, *PpF3H*, *PpF3’H*, *PpDFR1*, *PpANS*, *PpUFGT*, and *PpMYB10.1*/*2*/*3*), with the total number ranging from one to seven for each promoter (Figure 7A). For example, five G-box motifs were identified in the *PpCHS1* promoter, four G-box motifs, and one ACE motif were identified in the *PpMYB10.1* promoter, while only one G-box motif and one ACE motif were identified in the *PpANS* promoter (Figure 7A). To validate the effect of PpHY5 on the transcription of anthocyanin biosynthetic genes, the promoter sequences of 11 genes were isolated and the dual-luciferase assay was conducted. When co-infiltrated with *PpHY5*, the highest activity was observed in the promoter of *PpCHS1* (5.3-fold) followed by *PpMYB10.1* (4.9-fold), *PpCHS2* (4.1-fold), and *PpDFR1* (3.3-fold) (Figure 7B). However, no obvious enhancements in luciferase enzyme activities were detected for other genes. Similarly, infiltration of *PpHYH* was also able to activate the promoters of both *PpCHS1* (2.9-fold) and *PpDFR1* (2.6-fold), while no obvious change in activation was observed on the promoters of either *PpCHS2* or *PpMYB10.1* or other genes (Figure 7B). Compared with PpHY5, PpHYH showed a similar but weaker ability to activate the promoters of both *PpCHS1* and *PpDFR1*. Taken together, these findings indicated that PpHY5 functioned as a positive transcriptional regulator for anthocyanin accumulation via activation of anthocyanin biosynthetic genes, possibly via binding directly to their promoters at G−box and ACE motifs.

We also analyzed the effects of PpHY5 and PpHYH on the promoter activity of *PpHY5* itself. PpHY5 and PpHYH significantly stimulated *PpHY5* promoter activity with the ratio of LUC to REN of 6.6 and 4.3 respectively (Figure 7C). To investigate the specificity of the interaction between PpHY5 and the *PpHY5* promoter, we performed a combination of *cis*-regulatory element mutations and the dual-luciferase assays to compare the activation levels. Four predicted E-box motifs were found in the promoter of *PpHY5* and designated as E-box-1, E-box-2, E-box-3 and E-box-4 respectively (Figure 7D). As shown in Figure 7E, when E-box-2 (CAAATG) within the *PpHY5* promoter was mutated to ACAAGT, the trans-activation activity of *PpHY5* on *PpHY5*-m2 promoter was attenuated by 74%. In contrast, PpHY5 still showed similar activation towards other mutated *PpHY5* promoters (*PpHY5*-m1, *PpHY5*-m3 and *PpHY5*-m4) with the induction of 5.6-, 7-, and 6.4-fold, with no significant difference relative to the native *PpHY5* promoter (Figure 7E). These results suggested that PpHY5 might recognize E-box-2 to specifically activate its own transcription.

## Discussion

### UVA and UVB Increased Anthocyanin Accumulation in Peach by Up-Regulating the Expression of Anthocyanin-Related Genes

Peach is an economically important fruit crop cultivated worldwide. Fruit coloration is one of the critical traits of exterior quality and commercial value for the consideration in consumer choice. Anthocyanin is the predominant component responsible for red pigmentation in peach fruit (Cheng et al., 2014). Light is an essential environmental factor influencing the production of anthocyanin and fruit coloration in peach, indicating the light signaling pathway is involved in the process (Zoratti et al., 2014). In this study, we conducted the comprehensive analysis of the effects of different UV lights on anthocyanin accumulation in peach. The results showed that peach fruit accumulated anthocyanin under UVA or UVB treatment and the induction was strengthened by the irradiation of UVA and UVB together (Figure 1). The induction was due to enhanced expression of genes encoding enzymes of the anthocyanin biosynthetic pathway.

The effects of UV irradiation on anthocyanin accumulation were not apparent until after continuous UV light treatment for 72 and 144 h. However, the transcript levels of multiple genes were induced in a short time (Figure 2). The results were similar with the significant elevation in the transcripts of *AtCHS*, *AtF3H*, and *AtDFR* in Arabidopsis under blue light treatment for 1 h (Cominelli et al., 2008). Maximum transcriptional response was observed following irradiation with of UVA + UVB, which correlated with anthocyanin accumulation. The molecular evidence of the synergistic effect of UVA + UVB on the regulation of anthocyanin biosynthesis needs further study.

### The Function of PpHY5 Is Conserved in Different Light Signaling Pathways for Anthocyanin Accumulation

HY5, a member of the bZIP family transcription factor, is an essential regulator in multifaceted developmental processes, such as photomorphogenesis, pigment accumulation, chloroplast development, nutrient assimilation, and carbon/nitrogen balance (Gangappa and Botto, 2016). HY5 has also been functionally characterized to be involved in the regulation of anthocyanin accumulation in response to light in a variety of plants (Shin et al., 2013; Jiang et al., 2016; An et al., 2017). After exposure to blue light, the transcript level of *SlHY5* in tomato was induced sharply within 3 h, and the SlHY5 protein accumulated steadily and peaked at 48 h (Liu et al., 2018). In peach, *PpHY5* had a strong response to both UVA and UVB at the transcription level, and the expression rapidly increased within 6 h. Expression of *PpHYH*, the closest homolog of *PpHY5*, was also induced by UVA and UVB treatments. Meanwhile, a decrease in the transcription level of *PpCOP1.1* was observed.

In Arabidopsis under white light, the *hy5* mutant exhibited skotomorphogenic phenotype with less anthocyanin content and longer hypocotyl length, conversely the *cop1* mutant presented photomorphogenic phenotype with higher anthocyanin content and short hypocotyl length. Overexpression of *PpHY5* in *hy5* and *PpCOP1.1* in *cop1* restored wild type phenotypes under white light (Figure 4A). Moreover, the RT-qPCR analysis showed that anthocyanin biosynthetic structural genes, including *AtPAL*, *AtCHS*, *AtCHI*, *AtF3H*, *AtDFR*, *AtANS*, and *AtUFGT*, were significantly up-regulated in *PpHY5*/*hy5* and down-regulated in *PpCOP1*.*1*/*cop1* transgenic lines than those in the corresponding mutant (Figure 4C). The phenotypes in Arabidopsis transgenic lines and mutants implied that *PpHY5* and *PpCOP1.1* acted as counterparts. Under WWL, *PpHY5* also directly participated in UVA- and UVB-induced anthocyanin accumulation which depended on *PpCRY1* and *PpUVR8.1* photoreceptors, indicating that PpHY5 exhibited a positive regulatory role in anthocyanin biosynthesis in response to various wavelengths of light environment.

**FIGURE 8:**
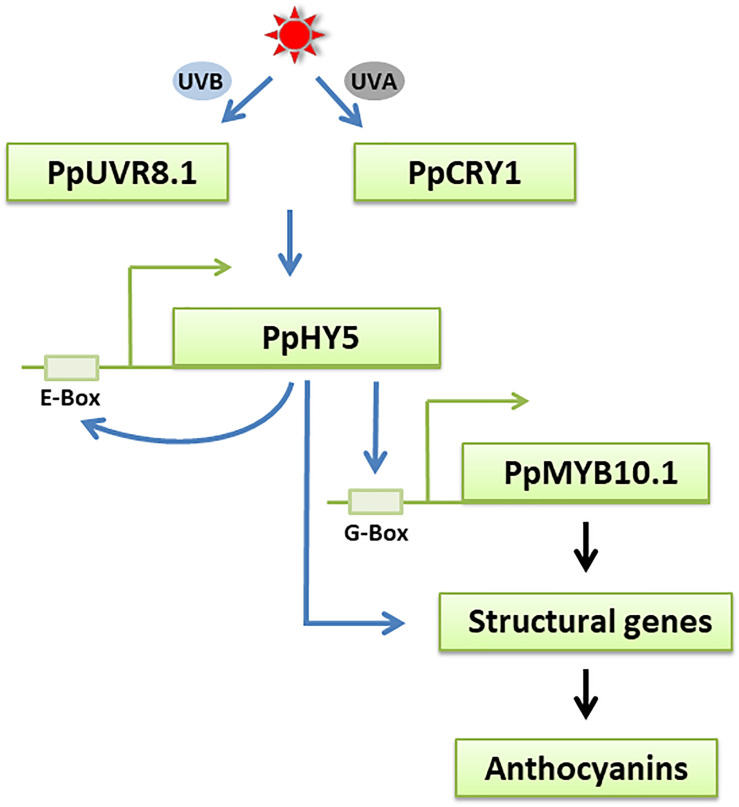
A possible model for UVA- and UVB-induced anthocyanin biosynthesis in peach fruit. The UV light receptors PpCRY1 and PpUVR8.1 respectively perceive UVA and UVB light signals and influences the expression of *PpHY5*, an essential regulator in UVA and UVB signaling pathway. PpHY5 positively induces its own transcription by interacting with an E-box in its own promoter and activates the expression of *PpMYB10.1* to regulate anthocyanin accumulation. The transcription of anthocyanin biosynthetic structural genes is directly regulated by *PpMYB10.1* and some of them were also regulated by *PpHY5*. The blue arrows indicate the pathways verified in the present work. The black arrows indicate the pathways that have been previously reported in peach.

We further investigated the role of PpHY5 in the regulation of anthocyanin accumulation using the dual-luciferase assay. PpHY5 positively activated the transcription of *PpCHS1*, *PpDFR1*, *PpCHS2*, and *PpMYB10.1* (Figure 7B). This is similar to results seen in tomato, apple, and pear (An et al., 2017; Liu et al., 2018; Tao et al., 2018). We also found that PpHYH had a similar effect to PpHY5 in regulating anthocyanin accumulation (Figure 7B). In addition, PpHY5 could bind to the E-box to regulate its own transcription (Figure 7E). As hypothesized, UVA and UVB light signals for anthocyanin biosynthesis were via PpHY5 in peach (Figure 8). These findings suggested that PpHY5 had a conserved function across different species and various wavelengths of light in regulating anthocyanin accumulation.

### PpCRY1 and PpUVR8.1 Contributed to UVA and UVB Signaling Pathways in Peach

Many studies have revealed that the photoreceptors are indispensable for anthocyanin biosynthesis. *VviUVR1* and *MpUVR8*, the UVB receptors in grape and liverwort, played a predominant role in the UVB-induced flavonoid accumulation (Loyola et al., 2016; Clayton et al., 2018). The phenotypes of alleviated hypocotyl elongation inhibition and decreased anthocyanin accumulation were observed in the antisense *Brassica napus CRY1* (*BnCRY1*) transgenic plant (Chatterjee et al., 2006). In apple, cryptochrome genes *MdCRY1* and *MdCRY2* have been identified to regulate anthocyanin accumulation and *MdCRY2* also participated in the photoperiodic control of flowering (Li et al., 2013a,b). Overexpression of *SlCRY1a* led to higher anthocyanin accumulation in tomato (Liu et al., 2018) and *SlUVR8* mediated the expression of *SlCHS* in response to the UVB environment (Li et al., 2018). We cloned the *PpCRY1* and *PpUVR8.1* from peach and characterized their functions as UVA and UVB receptors by complementation of Arabidopsis mutants. In our study, overexpressing *PpUVR8.1* or *PpCRY1* in the corresponding transgenic line promoted the expression of *AtHY5* in response to UVA or UVB irradiation, respectively. The results provided evidences that *PpCRY1* and *PpUVR8.1* acted as the photoreceptors of UVA and UVB and were associated with eliciting the transcript accumulation of *HY5*.

In conclusion, our study showed that UVA and UVB accelerated anthocyanin biosynthesis in the peel of “Hujingmilu” peach. The functions of crucial genes and photoreceptors in UV light signaling transduction pathways from peach were clarified. The data suggested that PpHY5 was both involved in UVA- and UVB-induced anthocyanin accumulation via a relatively conserved signaling pathway in PpCRY1-dependent and PpUVR8.1-dependent manner, respectively (Figure 8). PpHY5 regulated anthocyanin accumulation by activating the expression of anthocyanin biosynthetic-related genes. However, the results were mainly based on transcriptional control, which still needs to be verified at the post-transcriptional level in future experiments. Additionally, it would be worthy to investigate whether a direct interaction or an indirect interaction containing other unknown factors exists between PpCRY1 or PpUVR8.1 and PpHY5.

## Data Availability Statement

The raw data were deposited in NCBI sequence read archive (SRA) under accession number PRJNA665861.

## Author Contributions

YZ carried out the experiment, analyzed the data, and drafted the manuscript. MC helped to provide the experiment materials. CZ and RJ helped to provide technical assistance. TM, HW, AA, KL-W, and CX were involved in the revision of the manuscript. CX initiated the project, designed the research framework, coordinated the study, and participated in writing the manuscript. All authors read and approved the final manuscript.

## Conflict of Interest

The authors declare that the research was conducted in the absence of any commercial or financial relationships that could be construed as a potential conflict of interest.

## Footnotes

1 http://bioinformatics.psb.ugent.be/webtools/plantcare/html/

## Supplementary Material

Supplementary Figure 1 Subcellular localization of PpHY5 and PpCOP1.1 in *Nicotiana benthamiana* leaves under white light. eGFP, GFP channel; Nucleus-RFP, transgenic *N. benthamiana* plants with red florescence in the nucleus; Bright-field, light microscopy image; Merged, merged image of the GFP and Bright channels. Scale bars indicated 20 μm.

Supplementary Table 1 Primers used for reverse transcription quantitative PCR analysis.

Supplementary Table 2 Primers used for plasmid construction. The restriction sites are underlined.
