# Supplementary figures and images for: The Photomorphogenic Transcription Factor PpHY5 Regulates Anthocyanin Accumulation in Response to UVA and UVB Irradiation
Source: Front Plant Sci. 2021 Jan 18;11:603178. doi: 10.3389/fpls.2020.603178 (PMC7847898; doi:10.3389/fpls.2020.603178)

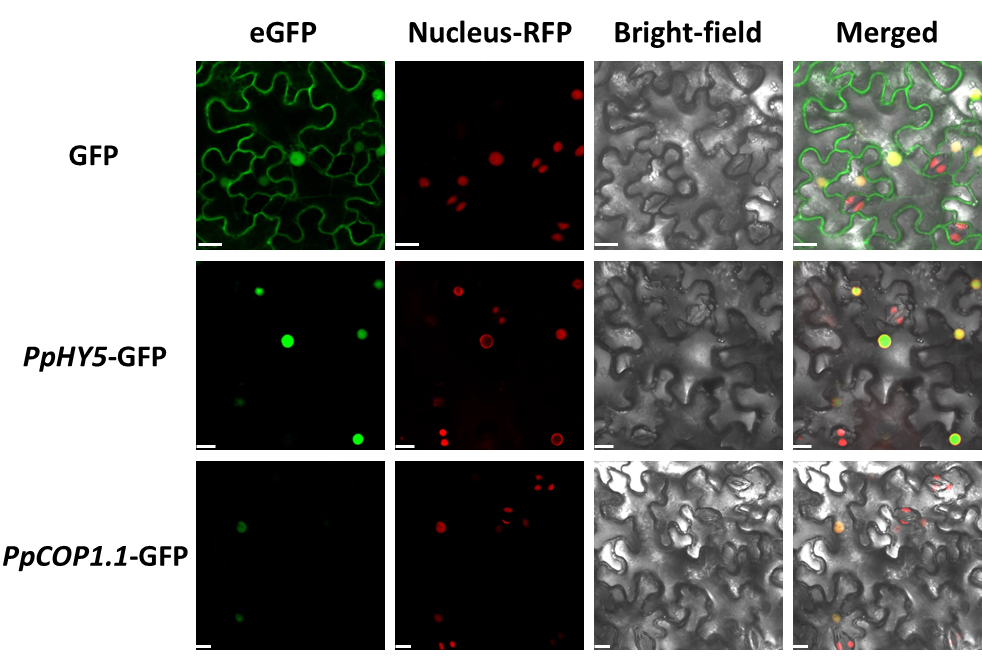

Supplement: Supplementary Figure 1 — Subcellular localization of PpHY5 and PpCOP1.1 in Nicotiana benthamiana leaves under white light. eGFP, GFP channel; Nucleus-RFP, transgenic N. benthamiana plants with red florescence in the nucleus; Bright-field, light microscopy image; Merged, merged image of the GFP and Bright channels. Scale bars indicated 20 μm. [file Image_1.TIF]
