# Supplementary material for: The Photomorphogenic Transcription Factor PpHY5 Regulates Anthocyanin Accumulation in Response to UVA and UVB Irradiation
Source: Front Plant Sci. 2021 Jan 18;11:603178. doi: 10.3389/fpls.2020.603178 (PMC7847898; doi:10.3389/fpls.2020.603178)
Supplement: Supplementary Table 1 — Primers used for reverse transcription quantitative PCR analysis. [file Table_1.DOCX]

**Table S1** **| Primers used for reverse transcription quantitative PCR analysis.**

| **Gene** | **Forward primer Sequence (5′ to 3′ )** | **Reverse primer Sequence (5′ to 3′ )** |
| --- | --- | --- |
| *AtPAL* | AGCAAATCCTGTGACGAACC | CGTTGACATGAGCTTGAGGA |
| *AtCHS* | CCTGACACATCTGTCGGAGA | GGAGATGGAAGGTGAGACCA |
| *AtCHI* | CGTGATCTTCACCGTCATTG | TTCACGGAAGAACGGGATAG |
| *AtF3H* | CTGATCTCACCCTCGGACTC | GATTGACGACAAACGCTCCT |
| *AtDFR* | TTCCTCTCATGATGCAACCA | TCCGTCAGCTTCTTGGAACT |
| *AtANS* | GGACAATTGGAATGGGAAGA | CTTCGCGTACTCACTCGTTG |
| *AtUFGT* | TTGATGGCAAGGAAATGTCA | GCTTCCTCCACACGAAACTC |
| *AtHY5* | GTTTGGAGGAGAAGCTGTCG | CTTCAGCCGCTTGTTCTCTT |
| *ATACT2* (Actin) | ATTCAGATGCCCAGAAGTCTTGTTC | GCAAGTGCTGTGATTTCTTTGCTCA |
