# Supplementary material for: The Photomorphogenic Transcription Factor PpHY5 Regulates Anthocyanin Accumulation in Response to UVA and UVB Irradiation
Source: Front Plant Sci. 2021 Jan 18;11:603178. doi: 10.3389/fpls.2020.603178 (PMC7847898; doi:10.3389/fpls.2020.603178)
Supplement: Supplementary Table 2 — Primers used for plasmid construction. The restriction sites are underlined. [file Table_2.DOCX]

**Table S2 | Primers used for plasmid construction.** The restriction sites are underlined.

| **Name** | **Forward primer Sequence (5′ to 3′ )** | **Reverse primer Sequence (5′ to 3′ )** |
| --- | --- | --- |
| *PpHY5*-SK | ATAAGAATGCGGCCGCATGCAAGAGCAAGCGACGAG | GGACTAGTCTATAAGGATCCTTCTGCATTTGC |
| *PpHYH*-SK | ATAAGAATGCGGCCGCATGTCAGTCCCCATCAAACCAG | GGACTAGTTTACTTAACTGATCCTGCCTTTGG |
| *PpCOP1.1*-SK | ATAAGAATGCGGCCGCATGGAGGAGTGCTCGACTGGGG | AACTGCAGTTAAGCAGCAAGAACCAGCACTTTA |
| *PpUVR8.1*-SK | ATAAGAATGCGGCCGCATGGCAGAAGAGGGAGCCAG | GGACTAGTTCAAAACCGTACGCGTTTCAC |
| *PpCRY1*-SK | ATAAGAATGCGGCCGCATGTCAGGTGGTGGATGCAGTGTAG | GGACTAGTTTACCCAGTTTGAGATAGCCGCCTC |
| *PpHY5*-GFP | GGGGTACCATGCAAGAGCAAGCGACGAG | GCGTCGACTAAGGATCCTTCTGCATTTGCAT |
| *PpCOP1.1*-GFP | GGGGTACCATGGAGGAGTGCTCGACTGGGG | GCGTCGACAGCAGCAAGAACCAGCACTTTAATG |
| *PpCHS1*-LUC | CGGGATCCGGATTTCATTCATTGAATAGAG | ATAAGAATGCGGCCGCCAGTGGCAGGACCCTCAGC |
| *PpCHS2*-LUC | CGGGATCCCATCTCTAGTGACCAAACACAA | ATAAGAATGCGGCCGCTTGCGAACTTCCTCGACGGT |
| *PpCHI*-LUC | CGGGATCCGGTCCGAGTGAGGTTGTTCTTGTTC | ATAAGAATGCGGCCGCGAAACGACGTCGCCTCGATC |
| *PpF3H*-LUC | CGGGATCCTGTACCAAACATTTAACCTTATCAA | ATAAGAATGCGGCCGCTTCATTGCTGAAGTTGTTGTAGG |
| *PpF3’H*-LUC | GCGTCGACACCCCTATAGTAAATCTCCTTTATG | ATAAGAATGCGGCCGCACGGTGATGAATATGAGAATAAACA |
| *PpDFR*-LUC | GCGTCGACTTATCCACGACCCTTCGACTTTG | ATAAGAATGCGGCCGCGATTCAGACTCTGGCCCCATATTT |
| *PpANS*-LUC | CGGGATCCGACAATTATGATTTGCAAGAAAGAC | ATAAGAATGCGGCCGCGATTGTTGCAATTCCACTGCT |
| *PpUFGT*-LUC | CGGGATCCGTGAAGTAAATATCATGCTCTT | ATAAGAATGCGGCCGCGATCATCATCAATCGGTTGTGGTG |
| *PpMYB10.1*-LUC | CGGGATCCCTTGGCTTTGATTATCTTTGTTC | ATAAGAATGCGGCCGCCATCTTCCTCTCTAGTCCAAGCTCC |
| *PpMYB10.2*-LUC | CGGGATCCGATAAGGACAGAACTATGGCC | ATAAGAATGCGGCCGCATCTTCCTCTCTAGTCCAAGCTCCT |
| *PpMYB10.3*-LUC | CGGGATCCTTAAGTTACGATGGCGGTAG | ATAAGAATGCGGCCGCCAAGCTCCTTTTTTCACATCCAA |
| *PpHY5*-LUC | CGGGATCCCATCACAACTGGTTGAGACTCGAG | ATAAGAATGCGGCCGCCGCTTGCTCTTGCATTTGG |
| *PpHY5m1*-LUC | TCCCAAAATCAAGAAAACCATTCACTTCCAGC | AATGGTTTTCTTGATTTTGGGAAAGCAATGAG |
| *PpHY5m2*-LUC | CACGCATACCTCAAATACAAGTAGATATTCAA | ACTTGTATTTGAGGTATGCGTGCGTGATTTCT |
| *PpHY5m3*-LUC | CCAAGTTCAAAATTGCCACAAGTTTAGAAACTG | ACTTGTGGCAATTTTGAACTTGGAGTATTTTCC |
| *PpHY5m4*-LUC | GTAAAGAGTTTGCTGTTACAGGTATTAAGAGCA | ACCTGTAACAGCAAACTCTTTACACTTTAATTA |
